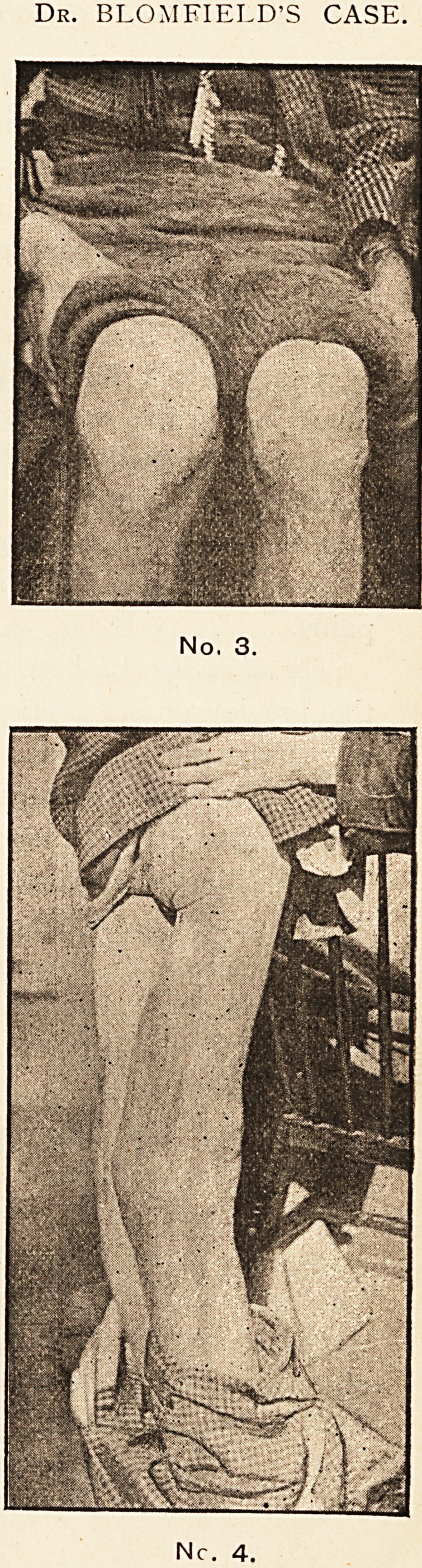# Two Cases of Locomotor Ataxy with Charcot's Joint Disease

**Published:** 1892-12

**Authors:** Henry Davy, Arthur G. Blomfield

**Affiliations:** Physician to the Devon and Exeter Hospital and to the Exeter Dispensary; Physician to the Devon and Exeter Hospital and to the Exeter Dispensary


### TWO CASES OF LOCOMOTOR ATAXY WITH CHARCOT'S JOINT DISEASE.

BY

HENRY DAVY, M.D. Lond.,

AND

ARTHUR G. BLOMFIELD. M.D. Aberd.,

*Physicians to the Devon and Exeter Hospital and to the Exeter Dispensary.*

---

For the reports and photographs illustrating these two interesting cases we are indebted to Mr. G. Stewart Abram, M.B. Cantab.

---

*Case I.* (under the care of Dr. Davy).—J. B., aged 53, was admitted to the hospital on August 11th, 1892, with the following history: He had passed most of his life in the merchant service, and dates the commencement of his troubles from an attack of gastro-enteritis, three years ago. Following this he had a girdle sensation, and eighteen months ago his left knee began to swell, and has painlessly increased in size since. There is a well-marked history of syphilis.

*On admission* the patient was found to be far from intelligent, and stated that he had come to have his knee operated upon. The condition of the knee is that shown in photograph No. 1. The whole knee, when the patient sits, feels tense and all the tissues are enlarged, with some increase of arthritic fluid. When the patient attempts to walk, which he is in the habit of doing with a crutch, there is a remarkable luxation of the femur and tibia. The line of the femur is altered, and the external condyle apparently wholly or in part destroyed, allowing the head of the tibia to be displaced inwards, as in a case of marked genu valgum. (See photograph No. 2.) There is absolutely no pain, and the right knee is unaffected.

The patient has many of the well-known symptoms of tabes dorsalis: ataxia and inco-ordination; speech and intelligence altered; knee-jerks completely gone; girdle sensation; some anæsthesia of lower limbs; bladder crises, shown by occasional attacks of retention and formerly involuntary micturition.

The pupils are not very characteristic, except that they are both small. They are unequal, and fail to react to either accommodation or light. The superficial reflexes are considerably increased.

*Case II.* (under the care of Dr. Blomfield).—John B., aged 48, curiously enough, came to the hospital on the same day as the previous case, and was admitted on August 13th, 1892.

Dr. DAVY'S CASE.

No. 1.

No. 2.

Dr. BLOMFIELD'S CASE.

No. 3.

Nc. 4.

The following was the history: He was in the army twenty-one years, and left it ten years ago. Twenty-six years ago he had a spear-wound in the chest, and four years ago an attack of right facial paralysis, due, he thinks, to cold. There is no definite history of syphilis. Eighteen months ago he first noticed symptoms of the present disease. He had difficulty in walking and weakness in the left leg, with well-marked lightning pains. Some four or five months before admission, owing to his inco-ordinate walk, he fell while trying to get into an omnibus, and from that time he noticed that his right knee was much worse and began to swell. Whilst in London he was suspended, and since then has lost his lightning pains.

*On admission* he had many symptoms comparable with the previous case. His intelligence was low, and his ataxia was most marked. The enlargement of the right knee was very apparent, and there was also slight enlargement of the left knee. (See photograph No. 3.) The swelling was painless, and resembled the enlargement already described, but the luxation was different. There appears to be more softening than destruction; and while the line of the femur is altered, the tibia is displaced backwards, as in a case of hyper-extension. (See photograph No. 4.)

This case presents more markedly than the previous one the symptoms of tabes dorsalis, nearly all the recognised features being present. His inco-ordination and ataxia are most marked. He has altered speech and intelligence. His pupils are pin-point, and give the Argyll-Robertson reaction. The knee-jerks are quite absent, and he has some anæsthesia of the lower limbs, with delayed sensation. He has crises of various kinds, notably intestinal and vesical. And, in addition, he still has some facial paralysis on the right side.

*Remarks*.—During the last twelve years no such typical examples of tabetic arthropathy as the two cases here recorded have come under our observation at the Devon and Exeter Hospital. It is known that cases of this kind are occasionally mistaken for disease of a joint requiring amputation or excision, and it is certainly conceivable that such might be the case where the nervous symptoms are slight while the joint disease is very serious. Again, as M. Charcot long ago pointed out, the arthropathy is an *early symptom* in locomotor ataxy; and he very properly insists that the absence of pain and inflammation is a most important point in the diagnosis of all diseases of the joint caused by locomotor ataxy. Two cases very similar to those now recorded are reported by Dr. James Murphy of Sunderland.[1] As regards the bearing of syphilis as a possible cause of the joint disease, it will be observed that in the first case there is a well-marked history of syphilis, while in the second one it is doubtful, though the fact that the man had facial paralysis is possibly suggestive of an old syphilitic infection. Virchow opposes the view that the joint disease occasionally found in tabetic subjects is a *special* arthropathy different from all other joint affections. He has no doubt at all that the usual causes of joint affection—mechanical and thermal causes—are sufficient to explain the disease. In his opinion, a large proportion of cases assumed to be tabetic were plainly syphilitic, and there is no doubt that arthritis deformans is the disease to be kept the most in mind. In the description of syphilitic affections of the joints given by Berkeley Hill and Arthur Cooper in the second edition of

[1] *Brit. Med. Journ.*, 1886, vol. ii., p. 168.

their work on *Syphilis*, the extensive and painless dislocations so characteristic of the affection in locomotor ataxy are not mentioned at all as occurring even in *tertiary* affections, and the joint is described as fixed, contracted, or much limited in its movements. The temperature, too, is often raised, as pointed out by Dr. Duffin.[1]

## Figures and Tables

**No. 1. No. 2. f1:**
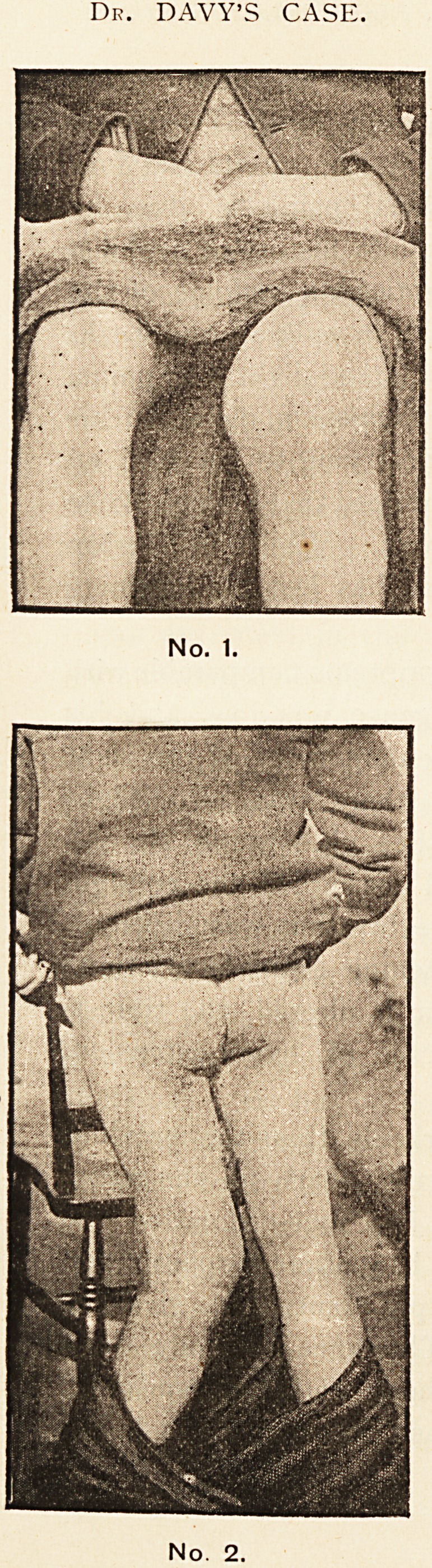


**No. 3. No. 4. f2:**